# A rare case of lymphangioma of the scrotum in a 3 year old boy: a case report

**DOI:** 10.1186/1757-1626-2-183

**Published:** 2009-11-03

**Authors:** Talal Al-Jabri, Anna Maria Gruener

**Affiliations:** 1 Department of Surgery, Royal Sussex County Hospital, Eastern Road, Brighton BN2 5BE, UK; 2 Moorfields Eye Hospital NHS Foundation Trust, Moorfields at St George's Hospital, Blackshaw Road, SW17 0QT, UK

## Abstract

Cystic hygromas, also known as lymphangiomas, are unusual congenital malformations of the lymphatic system. They are normally seen in the head and neck region and very rarely occur in the scrotum. This anomaly manifests as a painless scrotal swelling and is easily misdiagnosed. We report a rare case of a 3-year-old boy who presented with a gradually enlarging, painless scrotal mass which was identified sonographically and histologically as a scrotal lymphangioma and treated by surgical excision. A brief review of the literature is included.

## Introduction

Lymphangiomas are benign non-encapsulated lesions composed of sequestered non-communicating lymphoid tissue which is lined by lymphatic endothelium. They are subclassified by increasing vessel size, into (i) capillary, which is rare and located in the subcutaneous tissue, (ii) cavernous (located around the mouth and tongue), and (iii) cystic. Cystic hygromas show a predilection for the neck (75%) and maxilla (20%), with the remaining 5% arising in rare locations such as the mediastinum, retroperitoneum, bone, kidney, colon, liver, spleen and scrotum [1]. Scrotal lymphangioma presents as an unusual cystic scrotal mass. Misdiagnosis has been common. However, an awareness of the characteristic features of this lesion should lead to the correct preoperative diagnosis.

## Case presentation

**Figure 1:**
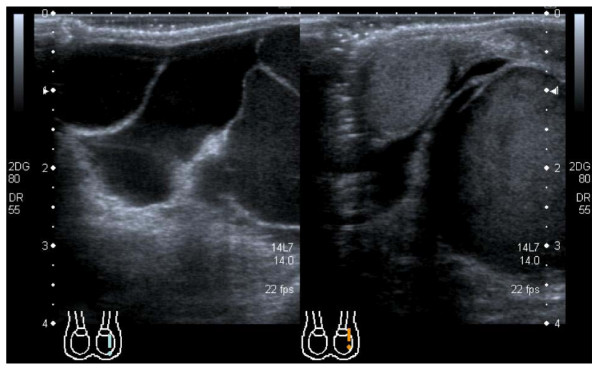
**The scrotal ultrasound scan demonstrates the multilocular cystic structure expected in scrotal lymphangioma**.

A 3-year-old Caucasian boy presented with a 4-month history of a gradually enlarging, painless swelling in his left scrotum. Scrotal examination showed retractile testes of normal size. The left side of the scrotum was visibly enlarged with a bluish tinge, and contained a non-tender, soft swelling distinct from the testes. It was possible to palpate above the swelling, which did not transilluminate. In view of the indeterminate nature of the examination an ultrasound of the testes was done and this demonstrated a multilocular cystic structure containing both clear and echogenic fluid in the left scrotum. The testes appeared normal. These findings were consistent with either a cystic hygroma or a complex spermatocoele (Fig. 1).

**Figure 2:**
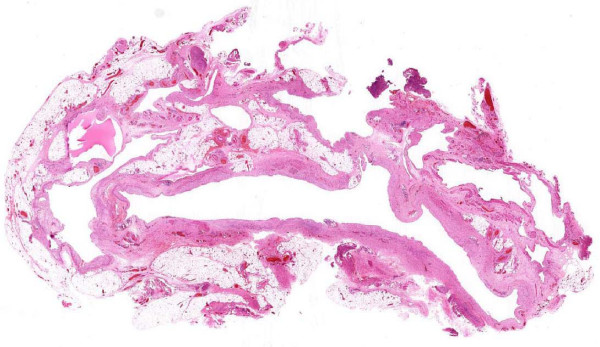
**Microscopic images showing dilated lymph channels**. The interstitium displays lymphoid cells and signs of fibroplasia.

A left inguinal scrotal exploration and excision of cystic swelling was performed. The left testis was 1.7 cm by 1 cm in size. The vas deferens had moderate-sized vessels associated with a large patent processus vaginalis. A multiseptate swelling separate from the left testis but infiltrating the scrotal skin was identified. Further surgical findings included a few separate cysts filled with haematoma. The swelling was dissected and excised from the adnexal tissues and histology revealed dilated lymph channels (Fig. 2).

Macroscopy showed dark red tissue measuring 3.7 cm by 2.3 cm by 1.0 cm and on slicing released blood. The cut surface showed a spongy texture but incorporated a central cyst 1 cm in diameter. Microscopy identified fibroadipose tissue surrounding a cavernous angiomatous malformation, consistent with a diagnosis of cystic hygroma. The parents were reassured of the findings. The postoperative recovery was unremarkable with complete resolution of symptoms.

## Discussion

Cystic hygromas, more widely known as lymphangiomas, are rare, hamartomatous, congenital malformations of the lymphatic system with no risk of malignant transformation. They mostly occur in childhood and have no sex or race predilection. Commonly found in the head and neck, they theoretically can occur anywhere in the skin or mucous membranes. Very rarely they can be found in the mediastinum, intestines, pancreas, mesentery, groin or bladder [1]. The intrascrotal occurrence of lymphangiomas is uncommon in children, especially when they do not relate to testicular structures [2]. The pathophysiology of lymphangiomas was first studied by Whimster in 1976 [3]. He identified the basic pathological process as a collection of primitive lymphatic cisterns in the deep subcutaneous plane, which fail to connect with the rest of the lymphatic system during their embryonic development. This is thought to be caused by congenital obstruction of lymphatic drainage.

In general, the diagnosis of a lymphangioma is based on the clinical history, physical examination and conventional light microscopy. The diagnosis of a scrotal lymphangioma (presenting as a painless, cystic mass), is confounded by multiple differential diagnoses including a hernia, hydrocoele, haematocoele, spermatocoele and varicocoele [4]. Rarely, a scrotal lymphangioma may present with acute scrotal pain (being mistaken for testicular torsion), cryptorchidism or lymphoedema of the thigh or scrotal skin, which may pose a diagnostic dilemma [4-6]. Probable preoperative misdiagnoses include hernia, hydrocoele, haematocoele, varicocoele, spermatocoele and torsion [7]. Histopathology shows numerous greatly dilated lymph channels in the upper dermis. These channels are filled with lymphatic fluid, but may contain erythrocytes, lymphocates, macrophages and neutrophils. The endothelial lining stains positive for Ulex europaeus agglutinin-I and the interstitium often shows lymphoid cells and evidence of fibroplasias [8].

Local recurrence is common and can only be prevented by complete surgical excision. Therefore, when suspected, imaging in the form of ultrasound and/or magnetic resonance imaging (MRI) is of critical importance to accurately define the extent of the lesion preoperatively, so an adequate resection may be performed in the first instance [9]. Lymphangiomas remain very rare entities. However an awareness of their existence should prevent their misdiagnosis, facilitating optimal management.

## Consent

Written informed consent was obtained from the patient for the publication of this case report and the accompanying images. A copy of the written consent is available for review by the Editor-in-chief of this journal.

## Competing interests

The authors declare that they have no competing interests.

## Authors' contributions

AMG and TAJ made substantial contributions to the analysis of data. AMG wrote the manuscript. TAJ edited the manuscript. AMG performed the literature search. All authors have approved the publication of this case report.
